# Retraction: Superior electrochemical performances of highly porous bismuth oxyhalide/lemon peel derived activated carbon electrode materials for solid state asymmetric and symmetric supercapattery devices

**DOI:** 10.1039/d6ra90026g

**Published:** 2026-03-02

**Authors:** Junaid Khan, A. Ahmed, Abdullah A. Al-Kahtani

**Affiliations:** a Department of Physics, Government Postgraduate College No. 1 Abbottabad Khyber Pakhtunkhwa Pakistan junaidkhan.nanotech@gmail.com; b Department of Higher Education Achieves and Libraries, Government of Khyber Pakhtunkhwa Pakistan; c School of Physics, Dalian University of Technology Dalian 116024 China; d Chemistry Department, College of Science, King Saud University P. O. Box 2455 Riyadh-22451 Saudi Arabia

## Abstract

Retraction of ‘Superior electrochemical performances of highly porous bismuth oxyhalide/lemon peel derived activated carbon electrode materials for solid state asymmetric and symmetric supercapattery devices’ by Junaid Khan *et al.*, *RSC Adv.*, 2025, **15**, 49565–49583, https://doi.org/10.1039/D5RA07844J.

The Royal Society of Chemistry hereby wholly retracts this *RSC Advances* article.

Junaid Khan had access to this material as a reviewer for another publisher and then submitted the same work to the Royal Society of Chemistry with a different author list. They have not been able to provide evidence that they carried out this work.

The authors were informed about the retraction of the article. Junaid Khan has not agreed with the decision, the other authors have not responded.

Signed: Laura Fisher, Executive Editor, *RSC Advances*

Date: 24th February 2026

